# Editorial: Novel immune markers and predictive models for immunotherapy and prognosis in breast and gynecological cancers

**DOI:** 10.3389/fimmu.2024.1431245

**Published:** 2024-05-29

**Authors:** Fuhao Wang, Qingyu Huang, Shicheng Guo, Alberto Traverso, Feifei Teng, Chao Liu

**Affiliations:** ^1^ Department of Radiation Oncology and Shandong Provincial Key Laboratory of Radiation Oncology, Shandong Cancer Hospital and Institute, Shandong First Medical University and Shandong Academy of Medical Sciences, Jinan, China; ^2^ Department of Medical Genetics, School of Medicine and Public Health, University of Wisconsin-Madison, Madison, WI, United States; ^3^ Department of Radiation Oncology (Maastro Clinic), School for Oncology and Reproduction (GROW), Maastricht University Medical Center, Maastricht, Netherlands; ^4^ School of Medicine, Libera Università Vita-Salute San Raffaele, Milan, Italy

**Keywords:** gynecological cancers, breast cancer, immunotherapy, precision medicine, biomarkers, prognosis

In the evolving field of oncology, precision medicine is reshaping treatment paradigms for breast and gynecological cancers by leveraging individual patient profiles (1–3). Utilizing advanced high-throughput sequencing technologies, researchers are identifying predictive biomarkers that improve the selection and efficacy of immunotherapies (4, 5). Despite significant advancements, clinical integration of these markers remains a challenge, underscoring the need for robust predictive models that combine multiple biomarkers to improve treatment precision and patient outcomes (6).

For this special Research Topic, we have gathered a collection of 15 research studies that focus on the discovery and use of new immune markers and predictive models in breast and gynecological cancers. The studies conducted by Ma et al. unveiled potential links between alterations in mitochondrial DNA methylation and the proliferative capacity of breast cancer cells. Their work in developing a prognostic model based on these mitochondrial DNA methylation dynamics offers valuable insights for predicting response to immunotherapy, assessing patient prognosis, and identifying new therapeutic targets. The meta-analysis by Wang et al. examined the prognostic implications of various PD-L1 expression patterns and the presence of tumor-infiltrating lymphocytes in high-grade serous ovarian cancer. Zhao et al. utilized both single-cell and bulk RNA sequencing to develop a predictive model based on B-cell marker genes for patients with triple-negative breast cancer. Yang et al. reviewed the multiple roles of liquid biopsy in the context of breast cancer immunotherapy, highlighting its utility in predicting and monitoring treatment outcomes, and identifying resistance mechanisms. The work by Zhang et al. highlighted the downregulation of *CYR61* in estrogen receptor-positive breast cancer and associated it with a poor prognosis. *CYR61*’s relationship with tumor suppressor pathways and its potential role in modulating the tumor immune microenvironment suggest its utility as a prognostic marker and therapeutic target. Zhou et al. identified reduced expression of *MSH6* as a marker potentially indicative of a favorable prognosis, and active immune landscape, and a better response to immune checkpoint inhibitors in endometrial cancer. Chen et al. proposed a prognostic model based on the expression of four genes (*RAD51AP1*, *HELLS*, *PLSCR4*, and *POLQ*) that could independently predict outcome and treatment response in breast cancer patients. Garcia-Torralba et al. suggest that a combination of immune biomarkers, genomic markers of proliferation (*AURKA* and *MYBL2*), and clinicopathological features can improve prognostic models for patients undergoing neoadjuvant therapy for breast cancer, potentially improving the management and stratification of early-stage disease. Yang et al. reported a case involving the use of an immunotherapy-based combination regimen to treat a patient with metastatic primary uterine sarcoma. Despite disease progression following multiple prior treatments, the patient experienced disease stabilization and partial remission after three rounds of combined immunotherapy, targeted therapy, and chemotherapy. The patient maintained a good quality of life, with prospects for long-term survival. Lv et al. found that serum levels of *BTNL8* may serve as a valuable prognostic tool for assessing outcomes in patients with high-risk human papillomavirus infection undergoing photodynamic therapy. This finding supports in the early screening and monitoring of disease progression in patients infected with high-risk human papillomavirus. Xiong et al. developed a prognostic model based on genes associated with programmed cell death. This model demonstrates excellent diagnostic performance and can predict clinical outcomes and levels of immune infiltration in high-risk endometrial cancer patients. Notably, *LRPPRC* is associated with poor prognosis, shows strong correlations with proliferative genes and several programmed cell death-related genes, and is highly expressed in patients with advanced clinical stages. Sammons et al. found that mutations in *B2M* and amplifications in *CD274* may help predict the therapeutic efficacy of immune checkpoint inhibitors in breast cancers with a high tumor mutational burden. This finding has implications for the targeted use of immune checkpoint inhibitors in the treatment of this subgroup of metastatic breast cancer patients. Zhao et al. demonstrated that detecting circulating tumor cells in breast cancer diagnosis is characterized by a sensitivity of 74% and a specificity of 98%. Furthermore, the presence of circulating tumor cell positivity is associated with worse overall survival and progression-free survival/disease-free survival in Asian populations. Zhang et al. provided a detailed review of the immunosuppressive tumor microenvironment in various molecular subtypes of endometrial cancer. They discussed the relationship between immune phenotypes and new immunotherapy strategies for advanced or recurrent EC, highlighting the potential for targeted therapeutic interventions based on molecular and immune classifications. Finally, Liu et al. developed and validated a nomogram that integrates ultrasonographic radiomic features, clinicopathological features, and ultrasound features. This model effectively predicts the likelihood of achieving a pathologic complete response in breast cancer patients undergoing neoadjuvant chemotherapy.

Through the papers included in this special Research Topic, we explore the importance and application of novel immune markers and predictive models in breast and gynecological cancers. These studies not only provide a deep understanding of the diversity of tumor immunology but also, through advanced sequencing technologies, reveal biomarkers for specific cancer subtypes and treatment responses, opening up new possibilities for personalized cancer treatment. While these studies highlight promising avenues, challenges remain. There is a need for larger and more diverse patient cohorts to validate the findings and improve the generalizability of the predictive models. Additionally, future research should focus on integrating multi-omics data with clinical information to develop robust and comprehensive predictive models. The integration of novel technologies, such as single-cell multi-omics, is likely to play a pivotal role in advancing our understanding of tumor biology and treatment response (7). We look forward to future research that will continue to make breakthroughs in this field and provide patients with more precise and effective treatment options.

## Author contributions

FW: Writing – original draft. QH: Writing – review & editing. SG: Writing – review & editing. AT: Writing – review & editing. FT: Writing – review & editing. CL: Writing – review & editing.
